# Tips and tricks for robotic pancreatoduodenectomy with superior mesenteric/portal vein resection and reconstruction

**DOI:** 10.1007/s00464-022-09860-0

**Published:** 2023-01-09

**Authors:** Emanuele F. Kauffmann, Niccolò Napoli, Michael Ginesini, Cesare Gianfaldoni, Fabio Asta, Alice Salamone, Allegra Ripolli, Armando Di Dato, Fabio Vistoli, Gabriella Amorese, Ugo Boggi

**Affiliations:** 1 Division of General and Transplant Surgery, University of Pisa, Via Paradisa 2, 56124 Pisa, Italy; 2 Division of Anesthesia and Intensive Care, Azienda Ospedaliero Universitaria Pisana, Pisa, Italy

**Keywords:** Pancreatoduodenectomy, Robotic pancreatoduodenectomy, Vein resection, Surgical technique, Tips and Tricks, Pancreatic cancer

## Abstract

**Background:**

Open pancreatoduodenectomy with vein resection (OPD-VR) is now standard of care in patients who responded to neoadjuvant therapies. Feasibility of robotic pancreatoduodenectomy (RPD) with vein resection (RPD-VR) was shown, but no study provided a detailed description of the technical challenges associated with this formidable operation. Herein, we describe the trips and tricks for technically successful RPD-VR.

**Methods:**

The vascular techniques used in RPD-VR were borrowed from OPD-VR, as well as from our experience with robotic transplantation of both kidney and pancreas. Vein resection was classified into 4 types according to the international study group of pancreatic surgery. Each type of vein resection was described in detail and shown in a video.

**Results:**

Between October 2008 and November 2021, a total of 783 pancreatoduodenectomies were performed, including 233 OPDs-VR (29.7%). RPD was performed in 256 patients (32.6%), and RPDs-VR in 36 patients (4.5% of all pancreatoduodenectomies; 15.4% of all pancreatoduodenectomies with vein resection; 14.0% of all RPDs). In RPD-VR vein resections were: 4 type 1 (11.1%), 10 type 2 (27.8%), 12 type 3 (33.3%) and 10 type 4 (27.8%). Vascular patches used in type 2 resections were made of peritoneum (*n* = 8), greater saphenous vein (*n* = 1), and deceased donor aorta (*n* = 1). Interposition grafts used in type 4 resections were internal left jugular vein (*n* = 8), venous graft from deceased donor (*n* = 1) and spiral saphenous vein graft (*n* = 1).

There was one conversion to open surgery (2.8%). Ninety-day mortality was 8.3%. There was one (2.8%) partial vein thrombosis, treated with heparin infusion.

**Conclusions:**

We have reported 36 technically successful RPDs-VR. We hope that the tips and tricks provided herein can contribute to safer implementation of RPD-VR. Based on our experience, and according to data from the literature, we strongly advise that RPD-VR is performed by expert surgeons at high volume centers.

**Graphical abstract:**

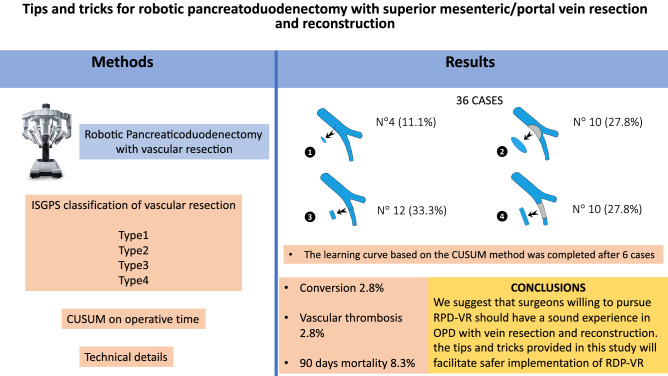

**Supplementary Information:**

The online version contains supplementary material available at 10.1007/s00464-022-09860-0.

After a long debate, and thanks to the availability of effective chemotherapy regimens, vein resection in pancreatectomy for pancreatic cancer is now considered standard of care [1, 2]. These operations are typically performed through an open approach that permits straightforward vascular reconstruction. Type of vein resection and reconstruction is classified according to the International Study Group of Pancreatic Surgery (ISGPS) [2]. In the open setting many vascular resections involve a vein segment and recostruction is frequently achieved by end-to-end anastomosis [3].

Recently, some groups have reported vein resection and reconstruction in minimally invasive pancreatoduodenectomy (MIPD) [4, 9]. However, even when using robotic assistance, which is known to facilitate intracorporeal sutures [10], most MIPDs with vein resection consist in small side-bite vascular resections that are often managed by an endoscopic stapler [4]. In MIPD other types of vein resection and reconstruction are feasible, but at the price of increased operative difficulty [5–9].

Involvement of the spleno-mesenteric junction is probably the most difficult scenario to manage in MIPD. Even when key collateral circulation is spared, ligature of the splenic vein results in severe sinistral portal hypertension in approximately one third of the patients. In open pancreatoduodenectomy (OPD), the splenic vein can be quite easily anastomosed to the left renal vein [3], while direct reconstruction of the superior mesenteric/portal vein (SMV/PV) is facilitated by either liver or intestinal mobilization [11]. All these maneuvers are difficult to implement in MIPD.

Our group has pioneered vascular resections in pancreatic cancer [12–14], having now performed over 700 open pancreatectomies with resection ± reconstruction. We implemented laparoscopic distal pancreatectomy in the late’90 s [15] and performed the first robotic pancreatoduodenectomy (RPD) in 2008 [16]. Thereafter, we have reported the feasibility of either vein [6] and artery [7] resections during RPD. Now, after some more experience, we wish to report on tips and tricks for RPD with vein resection (RPD-VR).

## Methods

### Selection criteria

Our selection criteria for RPD were previously reported [16, 17]. In general, as more experience was gained, we have gradually expanded our selection criteria. As specifically regards, RPD-VR, we still consider an absolute contraindication vein involvement ≥ 180° as well as segmental vein occlusion and/or vein thrombosis. However, in some patients vein involvement is not suspected until surgical exploration (unplanned vascular resection). In these patients, tumor abutment of the SMV/PV is typically limited. The decision whether to proceed with vein resection or to convert the procedure to open surgery is based on the possibility to proceed safely, while respecting the golden oncological principles established in OPD [12].

### Preoperative planning

Preoperative planning, based on careful review of computed tomography scans, is of paramount importance in MIPD. According to the recent expert consensus meeting on precision anatomy for MIPD [18], we carefully check the following items:

- 1.Presence of anatomical variations in arterial liver supply, and branching pattern of hepatic artery(ies);
- 2.Presence of anatomic variations in either superior mesenteric artery (SMA) and superior mesenteric vein (SMV) and their branching patterns;
- 3.Origin and course of inferior pancreaticoduodenal artery, dorsal pancreatic artery, first and second jejunal artery, first jejunal vein, left gastric vein, and inferior mesenteric vein;
- 4.Presence of celiac artery stenosis;
- 5.Presence of circumportal pancreas.

Vascular relationships between tumor and SMV/PV are also carefully noted, as previously described [19]. In addition, in preparation for triangle RPD [20] origin, course and branching pattern of right renal and adrenal arteries are also noted.

### Possible sources of vascular grafts

**Fig. 1:**
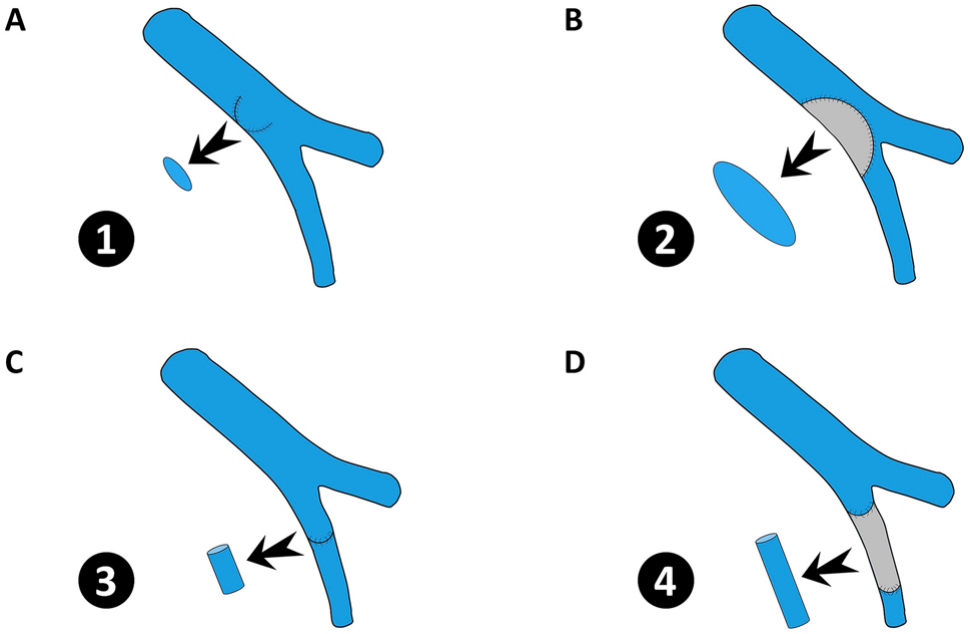
Types of vein resection and reconstruction according to the International Study Group of Pancreatic Surgery. **a**. Type 1 resection (small side-wall resection with direct repair); **b**. Type 2 resection (larger side-wall resection with patch repair); **c**. Type 3 resection (segmental resection with direct repair); **d**. Type 4 resection (segmental resection; an interposition graft is used for vascular repair)

Types of vein resection and reconstruction according to the ISGPS [2] are presented in Fig. 1.

Before trespassing the point of no return, a strategy for reconstruction must be established. Even when planning for direct repair (type 1 and type 3 resections) the surgeon must have a clear vision of which vascular grafts are available for possible vascular reconstruction. A full set of laparoscopic (or robotic) bulldog vascular clamps must be available, and the surgeon at the table and the scrub nurse must feel confident with their use.

Despite vascular prostheses have been successfully used to repair the SMV/PV [21, 22] we prefer to employ either autologous or allogeneic grafts. In case of type 4 vein resection our first choice is the internal jugular vein [23]. When this vein is not available, the next option is to use a spiral saphenous vein graft [24]. When neither these veins are available, we consider to use a graft from a deceased organ donor [25]. Additional sources of interposition grafts are the left renal vein [26], and the superficial femoral vein [27].

For patch repair (type 2 resection) we still prefer to avoid vascular prostheses. Autologous vascular patches can be obtained from the internal jugular vein, the greater saphenous vein [27], the inferior mesenteric vein [28], the right gonadal vein [29], the parietal peritoneum [30] and the falciform ligament [31]. Again, when these grafts are not available or are not suitable allogeneic grafts from deceased donors can be used. Bovine pericardial grafts can also be used as either tube grafts or vascular patches [4].

### Surgical technique

**Fig. 2:**
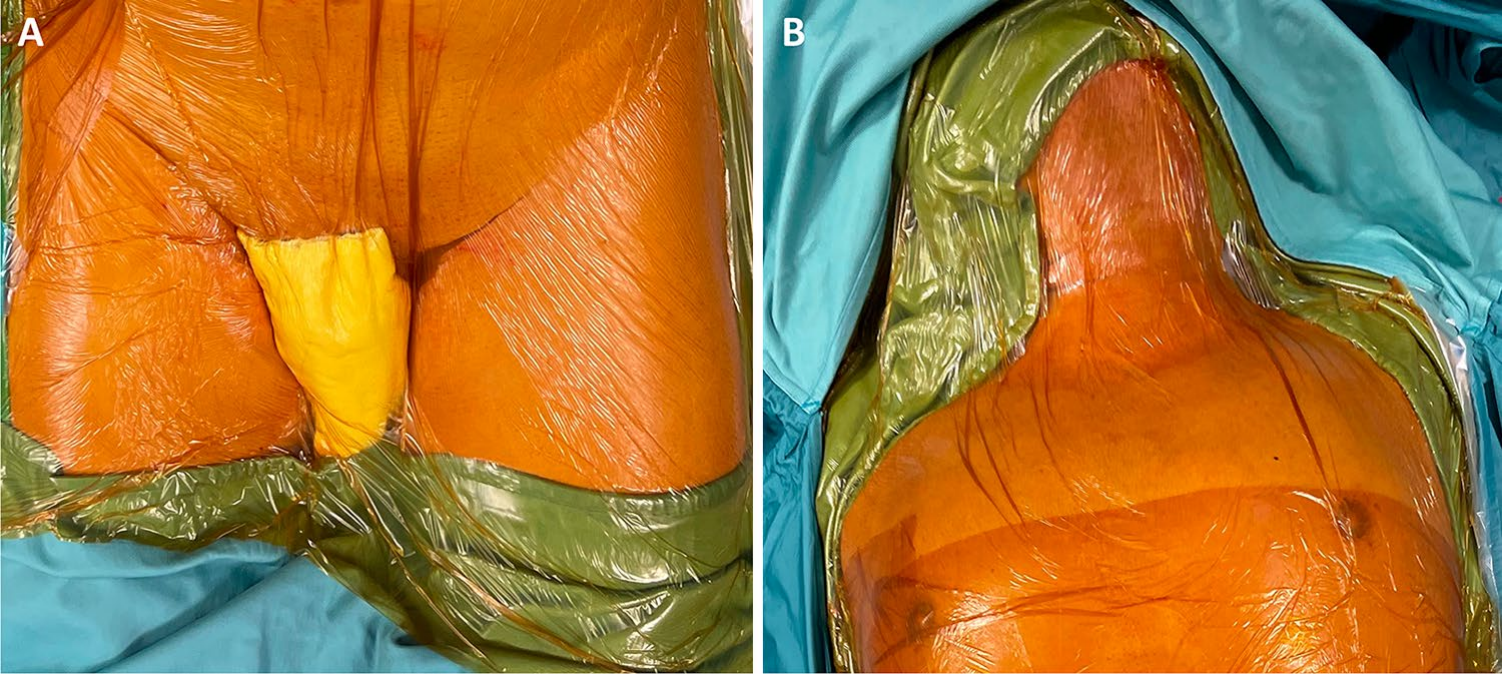
Groin (**a**) and neck (**b**) regions are prepped in preparation for possible harvesting of greater saphenous veins and/or left internal jugular vein

The patient is prepared as for a standard RPD [16, 20]. Part of this standard preparation includes exposure of both groins and the left lateral region of neck, in preparation for procurement of vascular grafts (Fig. 2).

**Fig. 3:**
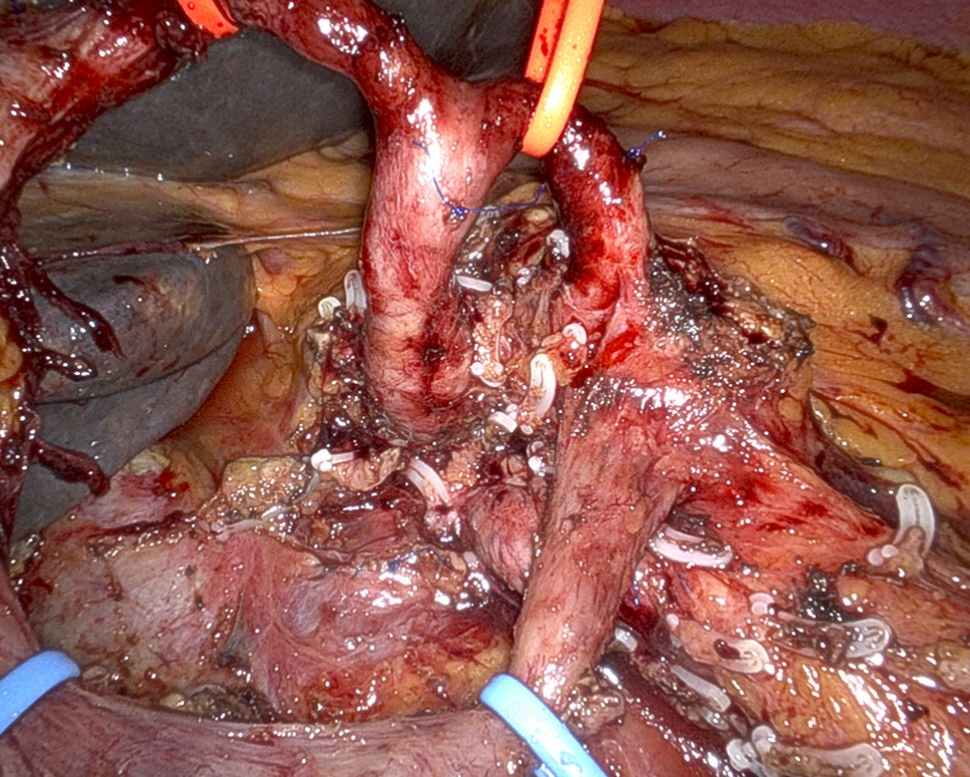
Following an “artery-first approach” with dissection in the periadventitial plane, the celiac trunk (with the origin of both the common hepatic artery and the splenic artery) and the right side of the superior mesenteric artery are radically divested

**Fig. 4:**
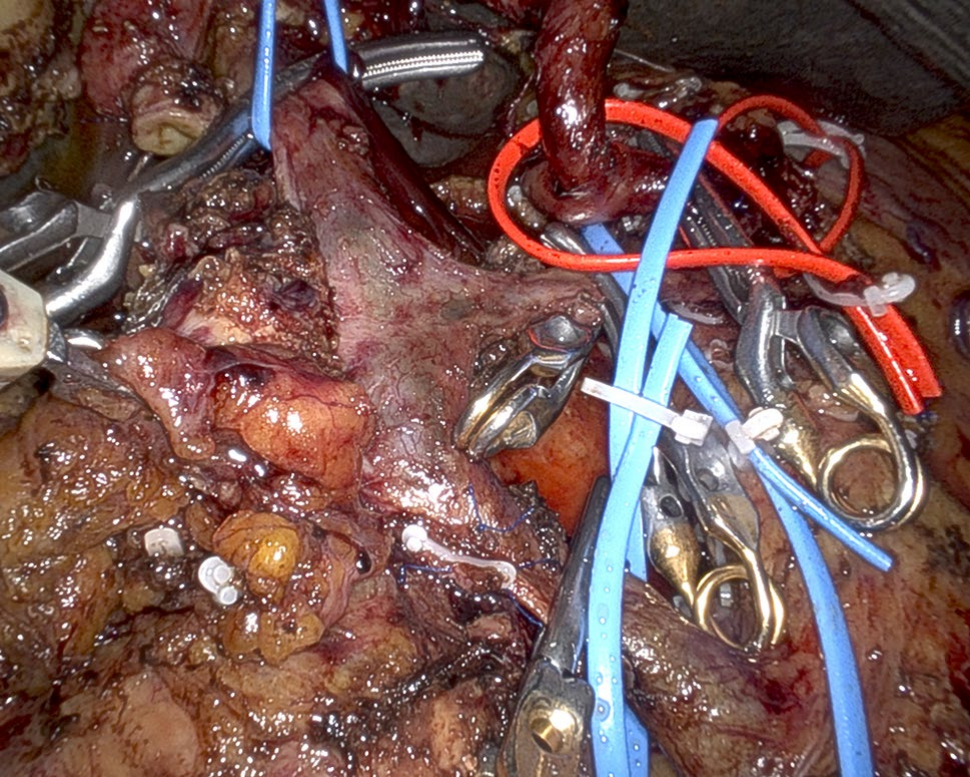
The specimen was mobilized from all retroperitoneal attachments, but the area of suspected vein involvement

Dissection proceeds following the technique recently reported for the “cold” triangle RPD [20]. The specimen is therefore mobilized from all retroperitoneal attachments, but the area of suspected vein involvement, by proceeding along the adventitial plane of large peripancreatic arteries (Figs. 3 and 4). At this time the surgeon has to decide which type of vein resection and reconstruction to perform.

**Fig. 5:**
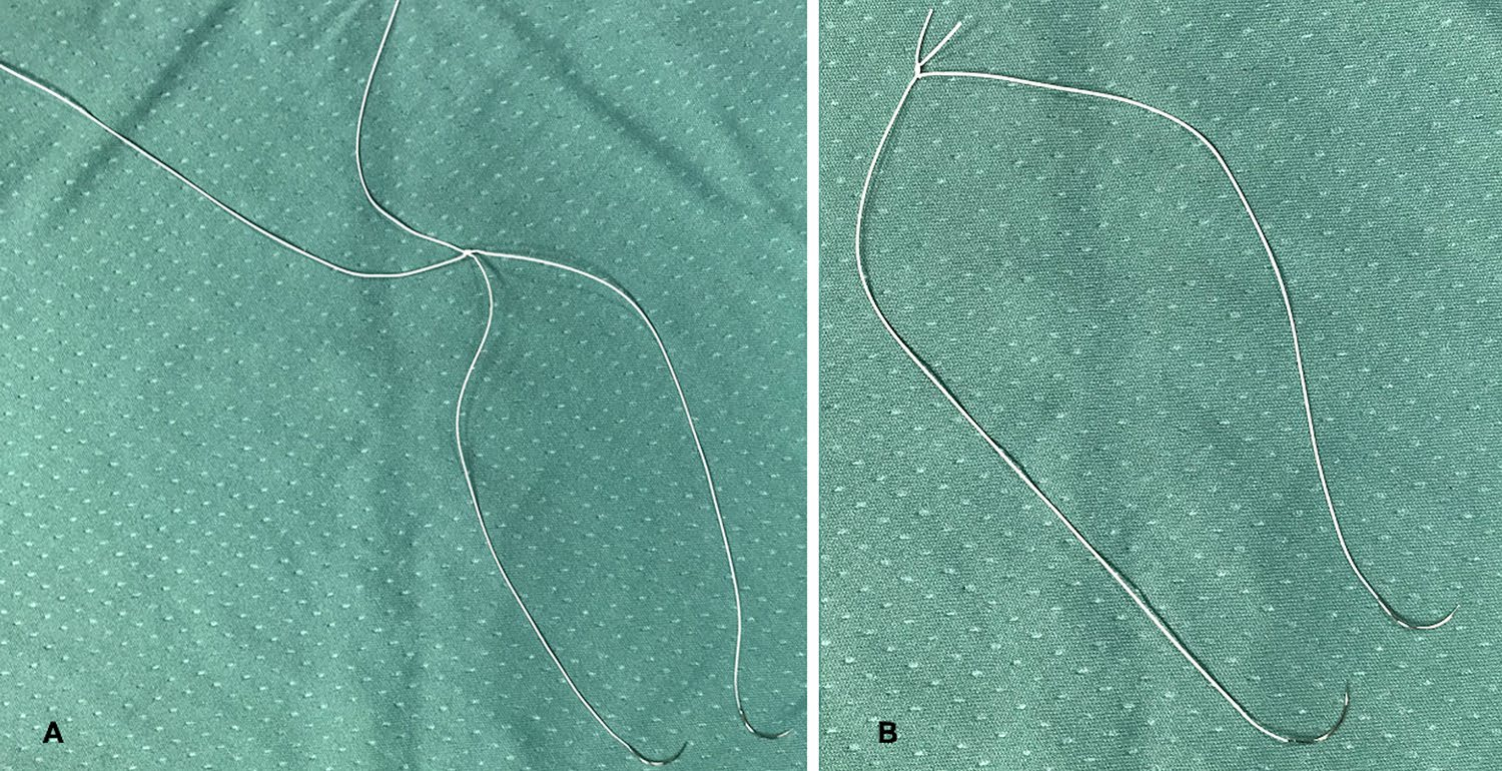
e-PTFE sutures are prepared to obtain double armed sutures of approximately 12 cm in length. **a**. Sutures tied together; **b**. Sutures ready for use

For running vascular sutures, we prefer to use expanded polytetrafluoroethylene (e-PTFE). To facilitate intracorporeal handling, two suture legs of approximately 12 cm each are tied together to obtain a shorter double armed suture (Fig. 5). Fine sutures are used (6/0 or 7/0).

The SMV/PV is crossclamped using laparoscopic bulldog vascular clamps. The SMA is also crossclamped, to reduce intestinal congestion during vein reconstruction.

After opening the vein, the vessel is flushed with topical sodium heparin injected through an 8 French Bracci ureteral catheter (Coloplast A/S, 3050 Humlebaek, Denmark) connected to a syringe handled by the surgeon at the table. Before completing the anastomosis, the vascular clamp on the SMV is released for a couple of seconds. Afterwards, the vein is flushed again with topical sodium heparin.

When reconstruction time exceeds 30 min, a bolus of unfractionated heparin (60–80 IU/kg body weight) is administered with the aim to increase activated clotting time between 200 and 250 s.

Upon completion of vascular reconstruction, in all types of vein resection, the first vascular clamp to be removed is the one on the portal vein. This maneuver permits to fix bleeding sites at a low pressure. Before removing vascular clamps, we place a small peanut sponge near to the SMV/PV. In case of bleeding the peanut sponge can be used for gentle vascular compression before hemostasis is secured by suture. The peanut sponge is made of oxidized regenerated cellulose. Therefore, it can be reabsorbed. This choice avoids the stressful situation, that might otherwise arise at the end of the procedure, if the (small) sponge was missing.

#### Type 1 vein resection and reconstruction

**Fig. 6:**
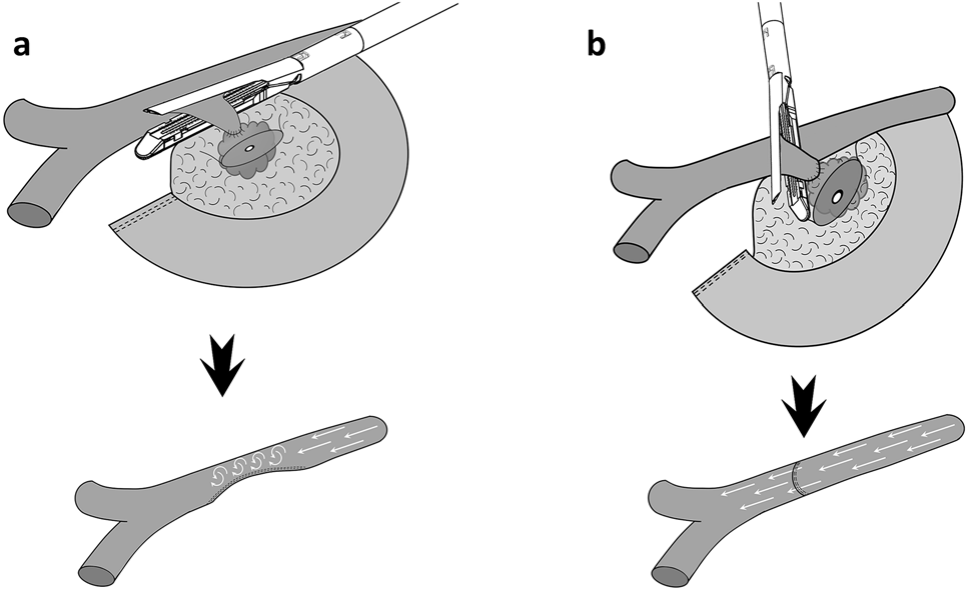
Stapled type 1 vein resection. **a**. Firing the endoscopic stapler parallel to the superior mesenteric/portal vein may create a vein stenosis, possibly leading to turbulence in portal flow; **b**. Firing the endoscopic stapler perpendicular to the superior mesenteric/portal vein decreases the risk of vein stenosis and therefore of turbulence in portal flow

Type 1 vein resection is performed when the area of tumor/vein contact is quite limited. In these patients a small side-wall vein resection is performed and vascular repair should not create a stenosis of the SMV-PV. This is why, when using an endoscopic stapler, the device should be fired perpendicular, rather than parallel, to the main axis of the SMV/PV. A multifire endo TA™ stapler (Covidien, Covidien IIc, Mansfiled, MA02048 USA) can be used to spare some vein wall. Alternatively, an endoscopic GIA stapler can also be used (Fig. 6). However, in RPD the short suture required in type 1 resections can be readily performed and the use of a stapler can be avoided (video 1).

#### Type 2 vein resection and reconstruction

**Fig. 7:**
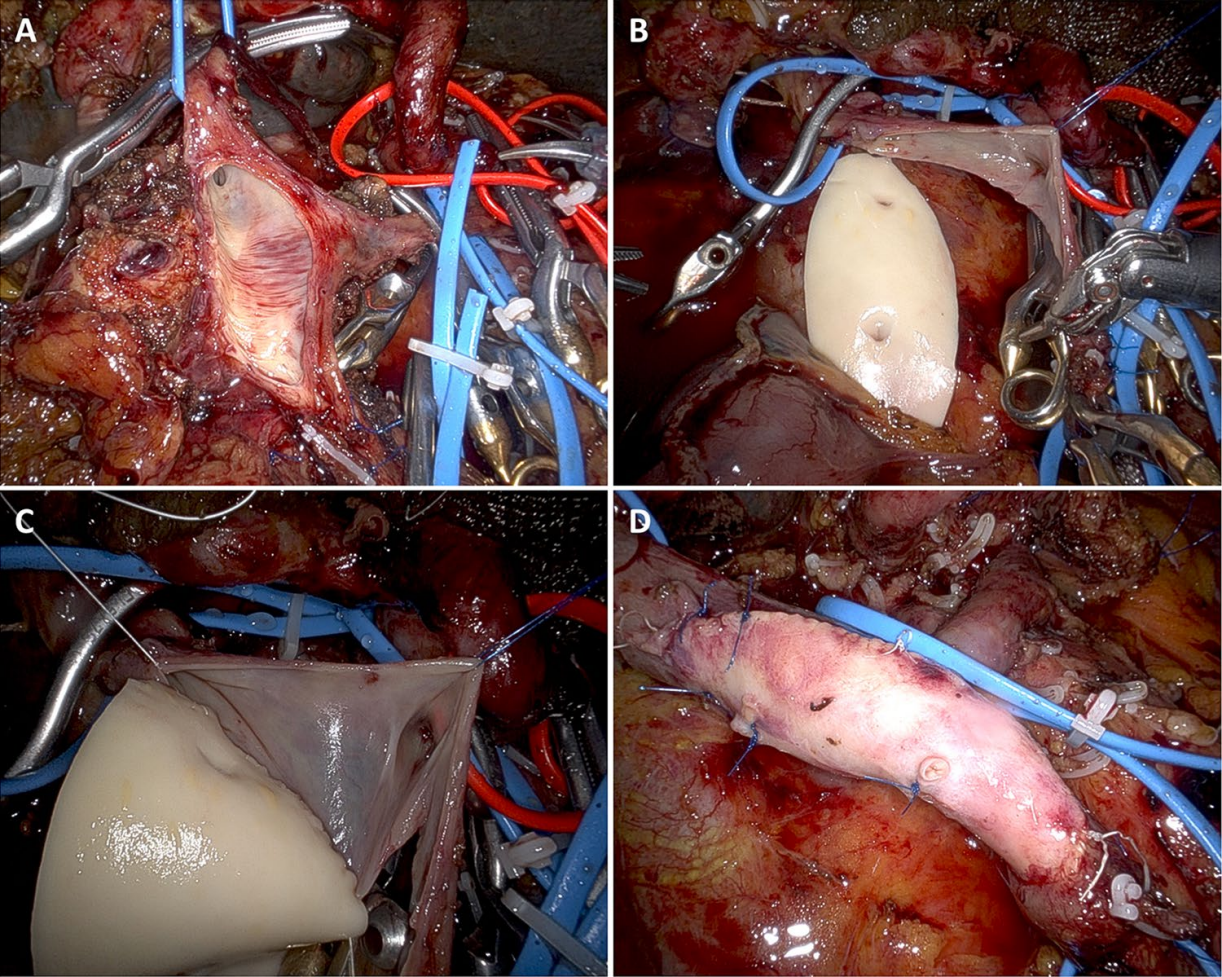
Type 2 vein resection. Repair is achieved using a large arterial patch from a deceased donor. **a**. Excision of a large vein patch en-bloc with the tumor; **b**. The arterial patch is placed near to the vein breach in preparation for suture; **c.** The posterior wall is sutured from inside. Note the optimal exposure achieved with the use of the arterial patch; **d**. Vascular repair completed

A type 2 resection is a larger side-wall resection. Primary closure is not possible and a vascular patch is required. In MIPD, type 2 resection may be particularly indicated when vein resection involves the spleno-mesenteric junction, because it avoids the need to anastomose also the splenic vein. Before proceeding to resection, the size of the patch must be carefully determined. To do so, we match the length of a suture to both longitudinal and transverse diameters of the side-wall vein resection. In RPD handling a large vein patch can complicate workflow. This is why, we prefer to use “more rigid” vascular patches, such as a deceased donor arterial patch. In all patients, a large vein patch is removed en-bloc with the specimen, paying careful attention to keep a safety margin from the area of suspected tumor invasion. A stay suture is placed on the anterior margin of the vascular defect to improve exposure. The posterior wall is sutured from the inside (Fig. 7) (video 2).

#### Type 3 vein resection and reconstruction

Before proceeding with type 3 vein resection and reconstruction in MIPD, the surgeon must decide if direct reconstruction is possible. However, for safety reasons, a plan for rescue conversion to type 4 reconstruction must be available.

**Fig. 8:**
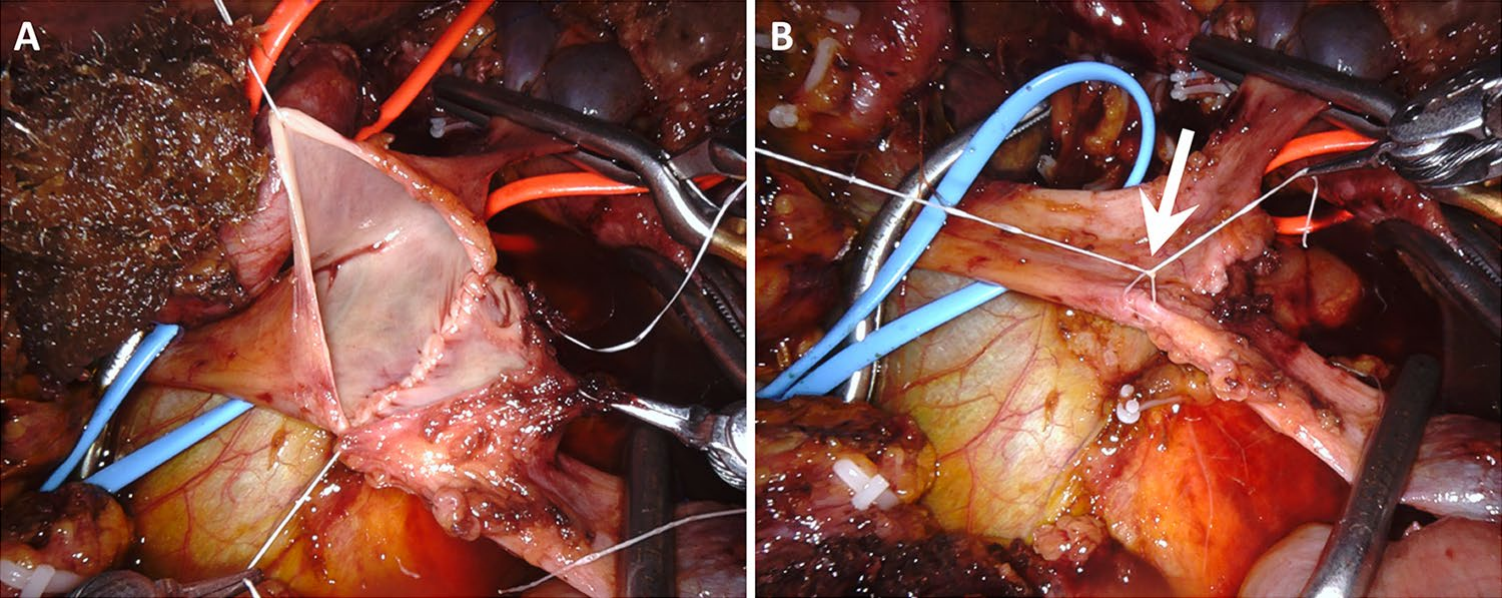
Type 3 vein resection and reconstruction. **a**. Completed posterior wall suture; **b**. At the end of the anastomosis, the two half-running sutures are tied by leaving a “growth factor”, aiming to minimize the purse-string effect that could otherwise result from the continuous suture

End-to-end vein anastomosis can be done using either two half-running sutures [32] or a parachute technique [33]. At the end, sutures must be tied with a growth factor, as originally described by Starzl. After clamp release migration of the extra suture ensures prompt expansion of the anastomosis thus reducing the risk of stenosis, that otherwise would occur from a purse string effect [32] (Fig. 8). To facilitate direct anastomosis, the degree of reverse Trendelenburg can be slightly decreased and a robotic instrument, holding a small peanut sponge, can be used to push the mesenteric root towards the liver. A type 3 resection is presented in video 3.

#### Type 4 vein resection and reconstruction

Type 4 vein resection and reconstruction is required when direct vascular reconstruction is not possible.

**Fig. 9:**
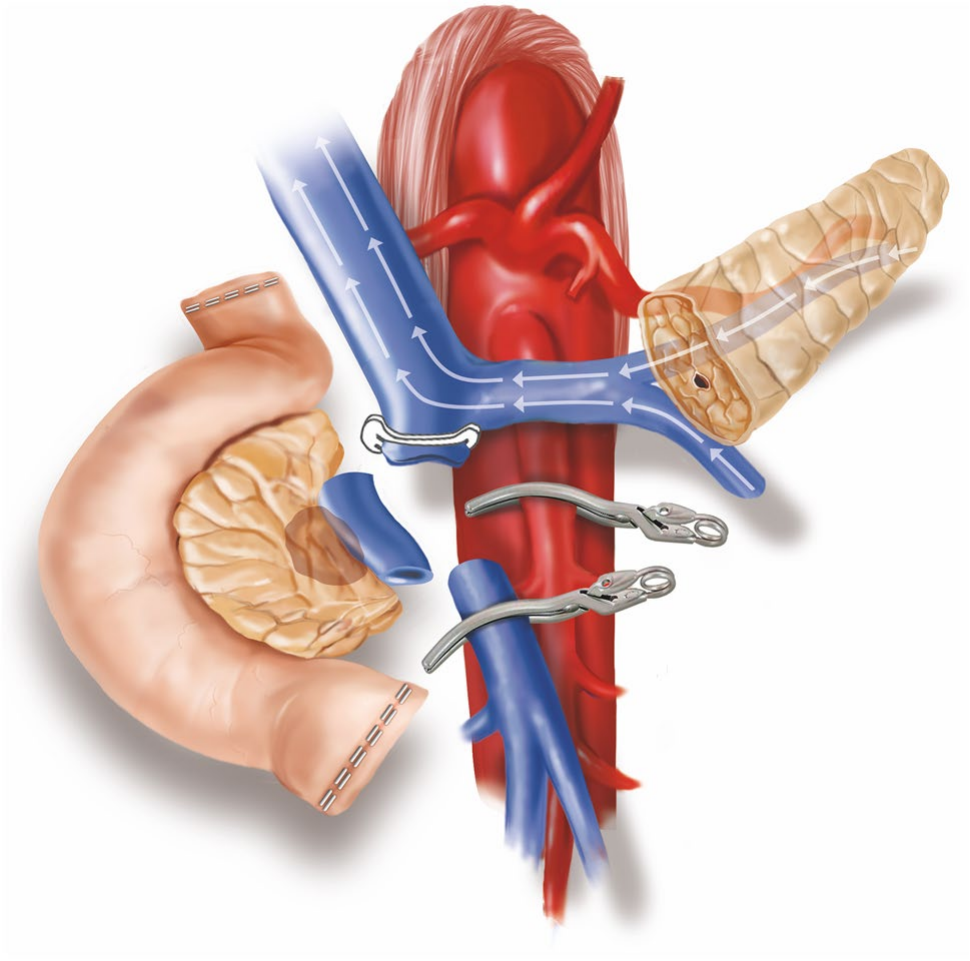
Type 4 vein resection and reconstruction. When resection involves the proximal portion of the superior mesenteric vein, portal flow can be maintained through the splenic vein (arrows). From Boggi U (Ed.) Minimally Invasive Surgery of the Pancreas. Springer-Verlag Italia s.r.l. 2018 – https://doi.org/10.1007/978-88-470-3958-2

**Fig. 10:**
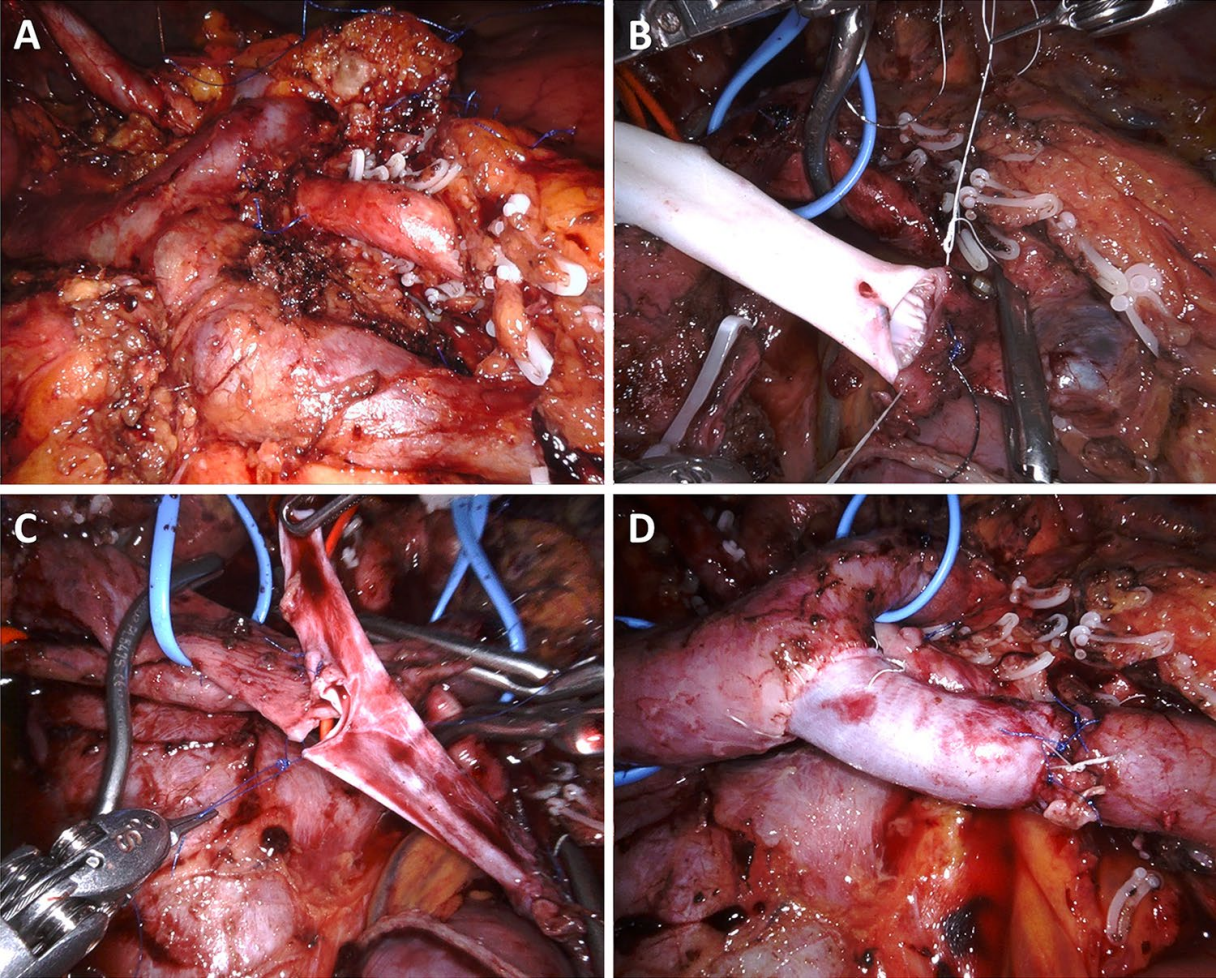
Type 4 vein resection and reconstruction. **a**. The specimen was detached from all retroperitoneal attachments but the area of tumor adhesion to the superior mesenteric vein; **b**. Proximal anastomosis between the interposition graft and the superior mesenteric vein; **c.** The interposition graft is trimmed in preparation for the distal anastomosis; **d**. Vascular reconstruction completed

Considering that two anastomoses are required, in type 4 vein resection and reconstruction initially only the SMV is crossclamped to maintain some portal flow through the splenic vein. Actually, if the inferior mesenteric vein drains into the splenic vein, also some venous outflow from the intestine may be maintained (Fig. 9). Once everything is ready for reconstruction the interposition graft is inserted into the abdomen. A suture is placed at the distal margin of conduit to facilitate graft orientation and handling. The anastomosis between the SMV and the interposition graft is performed first, using two half-running sutures as described for type 3 vein reconstruction. Next, splenic and portal veins are crossclamped, the SMV is divided near the spleno-mesenteric junction, the graft is trimmed at the appropriate length, and the second anastomosis is fashioned using the same technique described above (Fig. 10) (video 4).

### Anticoagulant prophylaxis and postoperative surveillance

Anticoagulation prophylaxis was not increased because of vein resection and reconstruction. According to our standard protocol, patients receiving chronic anti-aggregant or anticoagulant therapies are converted to low-molecular-weight heparin (LMWE) the week before surgery. The remaining patients receive the first dose of LMWE approximately 8 h before surgery. Postoperatively, LMWE is maintained for 4 weeks. No chronic anticoagulant or anti-aggregant therapy is prescribed only because of vein reconstruction.

### Learning curve for RPD with vein resection and reconstruction

Feasibility learning curve for RPD with vein resection and reconstruction was defined based on operative time and was assessed with cumulative sum (CUSUM) analysis. The turning point of operative time curvature was used as a cut-off to compare 90-day mortality before and after completion of the feasibility learning curve. The ODD ratio was considered appropriate as measure of the effect size. The level of significance was set at *p* < 0.05.

## Results

Between October 2008 and November 2021, a total of 783 pancreatoduodenectomies were performed at the Division of General and Transplant Surgery of the University of Pisa. Vein resection and reconstruction was performed in 233 patients (29.7%). Two hundred and fifty-six of these patients underwent RPD (32.6%), including 36 RPDs-VR (14.0%). Overall, RPD-VR was performed in 4.6% of all patients undergoing pancreatoduodenectomy, and in 15.4% of those requiring a vein resection. Concerning the type of vein resection, 4 were type 1 (11.1%), 10 were type 2 (27.8%), 12 were type 3 (33.3%) and 10 were type 4 (27.8%). Vascular patches used to repair type 2 resections were made of peritoneum (*n* = 8), greater saphenous vein (*n* = 1), and deceased donor aorta (*n* = 1). Interposition grafts used in type 4 resections were internal left jugular vein (*n* = 8), venous graft from deceased donor (*n* = 1) and spiral saphenous vein graft (*n *= 1).

Only one patient (2.8%) underwent conversion at the end of the procedure because of diffuse bleeding in a patient with previous bone marrow transplant and multiple comorbidities.

Mean operative time was 610 ± 85 min, with a median estimated blood loss of 878 ml (IQR: 680.4 -1430.2). Mean SMV/PV clamping time was 27.4 ± 17.6 min (type 1: 13.5 ± 1.3 min; type 2: 17.8 ± 1.4; minutes; type 3: 25.4 ± 1.7 min; type 4: 45.1 ± 5.3 min).

Ninety-day mortality was 8.3%. Excluding one death occurred during the learning curve, (procedure number 23), 90-day operative mortality was 5.5%. Both patients died due to delayed extraluminal hemorrhage. The first patient bleed from the SMA, without evidence of leak or pancreatitis. We speculate that hemorrhage resulted from too “deep” arterial divestment. The second patient developed a biliary leak, leading to erosion of both hepatic artery and SMV/PV. When the 36 RPD-VRs were divided in two groups of 18 procedures each, there were two deaths in the early experience (11.1%) and one thereafter (5.5%). However, the difference was not statistically significant (*p* = 1, Fisher exact test). Mortality at 90 days for contemporary, unmatched, OPD-VR was 5.1%.

There was 1 (2.8%) partial thrombosis of the reconstructed SMV/PV, treated with heparin infusion. One patient required repeat surgery due to “sentinel bleeding” secondary to partial erosion of a peritoneal patch caused by grade B postoperative pancreatic fistula.

**Fig. 11:**
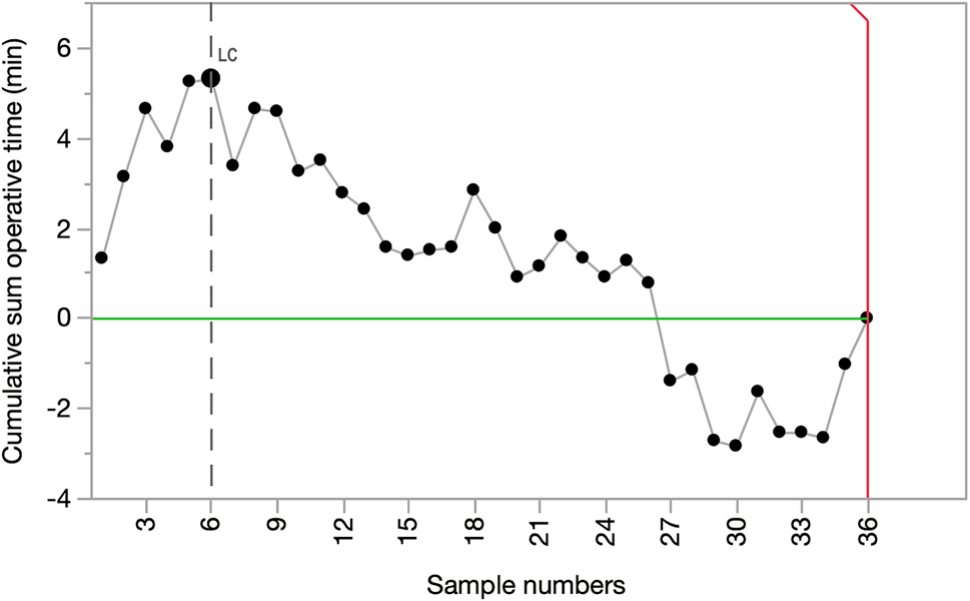
CUSUM chart accordingly to the operative time

Feasibility learning curve for RPD-VR was completed after 6 procedures (operative time declined from a median of 725 min [596.3–741.3] to 600 min [540–632.5]; p 0.027 (Fig. 11). After this turning point 90-day mortality declined from 33.3% to 3.3% [OR 14.5 (1.06–198.81); p = 0.039].

## Discussion

Patients undergoing RPD-VR at our institution were highly selected. MIPD with vein resection and reconstruction is a demanding procedure that should be accurately planned and carefully performed. Unexpected vascular involvement is among the leading causes of conversion to open surgery in MIPD [34, 35], and injury to the superior mesenteric vessels has been associated with few perioperative deaths [36, 37].

Approximately 30% of the patients diagnosed with pancreatic cancer have a borderline resectable tumor [38]. Following neoadjuvant treatments, if there is no disease progression and Ca 19.9 has dropped below 50% of the pretreatment level [39], borderline resectable pancreatic tumors should be resected en-bloc with the involved vein segment. However, in some patients, vascular involvement is discovered only during surgery [40]. These patients usually do not have major vein involvement and vascular resection and reconstruction can be performed through a minimally invasive approach. Therefore, surgeons willing to pursue MIPD should be aware of the need to manage unexpected vein involvement. If the surgeon does not feel comfortable in proceeding to RPD-VR, conversion to open surgery is required. Trying to spare a vascular resection by insisting in difficult perivascular dissections, could result in positive resection margins or could lead to massive hemorrhage requiring emergency conversion to open surgery. In a collaborative study, conversion to open surgery was required in 65 of 709 MIPDs (9.1%). Vascular involvement was the leading cause of conversion (18/65; 27.7%). Overall, there were 12 emergency conversions (18,4%). Emergency conversion was associated with increased intraoperative blood loss and a higher blood transfusion rate [34].

Few preliminary experiences showed that MIPD with vein resection and reconstruction is feasible and that side-wall resections are frequently employed [4–9]. However, conversion to open surgery may be required in as many as one third of the patients [41], and only few comparative studies have been published. Beane and coworkers compared 50 RPD-VR to 330 unmatched RPDs. RPD-VR had greater mean preoperative Ca 19.9, higher prevalence of pancreatic cancer, firmer gland texture, and more frequent administration of neoadjuvant therapies. Postoperative outcomes were similar, but there was a trend toward greater 90-day mortality in RPD-VR (8.0% versus 2.8%) [4]. Marino et al. compared 10 RPDs-VR to 73 unmatched RPDs. At the baseline the two groups differed for the more frequent delivery of neoadjuvant treatments in RPD-VR. RPD was associated with longer operative time, higher blood loss, and more frequent use of blood transfusions. Ninety-day mortality was comparable [42]. Jin and coworkers compared 14 RPDs-VR to 70 OPDs with vein resection. The two groups were comparable at the baseline and perioperative results were equivalent. However, 90-day mortality in RPD-VR was 7.1% (versus 4.3% in OPD) [43]. Yang and coworkers provided a propensity matched comparison between 13 MIPDs with vein resection (including 6 RPDs-VR) and 13 OPDs with vein resection. The only difference was longer operative time in MIPD (720 vs. 485 min). It is worth to note that 5 MIPDs required open vascular reconstruction and that 2 laparoscopic MIPDs had a robotic vascular reconstruction (53.8%). Only type 1 procedures were completed by the initial minimally invasive approach [44]. These data demonstrate the need for further studies, as already proposed by the Miami guidelines on minimally invasive pancreas resection [45]_._ While it could be still too early to plan for a randomized controlled trial, registry analysis should be the next step in providing meaningful comparison with the open procedure.

RPD-VR is a formidable operation raising the question of who should be allowed to do it. The starting point, is the annual volume threshold of 20 MIPDs recommended by the Miami international guidelines [45]. Considering that approximately 30–40% of patients may be eligible for MIPD, it appears that centers willing to implement RPD should perform an average of 60 PDs per year [46]. Despite only some 20% of the centers reach this cutoff [47], recent evidence confirms that ≥ 60 pancreatic resections are required to qualify as a high volume center and that results further improve when this yearly number of procedures is met [48]. It is therefore wise to advice implementation of RPD only at high volume centers that have reached clinical excellence in OPD. The need for prudent implementation of RPD-VR is further reinforced by the lack of comparative studies with the open procedure, and by the fact that only few centers have performed a good number of RPDs. A recent review demonstrated that up to march 2021 only 28 centers had reported on RPD [49]. Number of PDs per center ranged from 6 to 500. Eighteen centers reported ≥ 37 procedures, and therefore completed the initial learning curve [50, 51]. Only six centers reached the threshold of 100 RPDs, that was associated with truly improved postoperative outcomes [52]. Finally, despite the lack of studies on the impact of individual surgeon volume in RPD, experience with OPD shows that also surgeon volume is associated with improved outcomes [53]. Therefore, we strongly recommend that RPD-VR is implemented in centers with an annual volume ≥ 20 RPDs, following completion of the learning curve. In this context, RPD-VR should be performed by expert surgeons.

In this study, completion of feasibility learning curve for RPD-VR was achieved after 6 procedures and was associated with improved 90-day mortality. However, these figures should be carefully interpreted in light of some limitations. First, before performing the first RPD-VR we had already performed 22 RPD and 63 robotic pancreatic resections. Second, our group has one of the largest world experiences with vascular resection and reconstruction in open pancreatectomy. Third, we are also transplant surgeons. Fourth, we had contemporary practice with other robotic operations requiring intracorporeal vascular anastomosis. Therefore, the generalizability of these results remains to be established.

From a technical point of view not all vein resections are the same. Wedge resections are not expected to increase postoperative morbidity as opposed to segmental resections [54]. Type 1 resections are just minor variations of the standard technique. In MIPD the majority of type 1 resections are performed using a vascular stapler [4].

In OPD, vein reconstruction can be readily accomplished in most patients irrespective of resection type. In MIPD, vein reconstruction following segmental vein resection adds further complexity. First, the bowel cannot be fully mobilized and pushed towards the liver. In addition, the patient is in a reverse Trendelenburg position. Second, when using a jump graft, the surgeon must consider that at the end of the procedure the reverse Trendelenburg position will be abolished. This could create a vascular kinking, if the interposition graft was too long. Third, vascular control can be troublesome when the SMV is involved near the mesenteric root. In these patients, once the vein has been divided, repositioning the bulldog clamp on the SMV may be extremely difficult. Fourth, the entire team must be ready for emergency conversion in case of uncontrolled bleeding. Emergency conversion in robotic surgery should occur according to a standardized protocol [55]. Fifth, if the vein segment to be resected includes the spleno-mesenteric junction, management of the splenic vein creates additional challenges.

Our experience shows that segmental vein resection and reconstruction is feasible in RPD. However, in patients with tumor abutment ≤ 180° needing type 4 resections including the spleno-mesenteric junction a large type 2 vein resection and reconstruction could be preferable. In these patients, arterial patches would be the ideal choice because they improve exposure during the suture and provide good support to the reconstructed vein, potentially preventing angulation and collapse. Of course, availability of an arterial graft is a major limiting factor, especially when vein resection is not planned. Fresh deceased donor graft may be available only at transplant centers. Cryopreserved grafts can be ordered from a tissue bank, but usually not an urgent basis.

In our experience complexity of RPD-VR was shown by a mean operative time approaching 10 h and a median estimated blood loss of approximately 900 ml. Despite we have not provided a matched comparison with contemporary RPD, these figures exceed those that we have published for standard RPD [16, 17, 20]. Operative time and blood loss are well established quality metrics in PD [56]. In the best scenario, RPD-VR could achieve the same postoperative results of RPD. More realistically, RPD-VR is expected to increase the risk of postoperative complications when compared to RPD. The key question, however, is whether the burden of postoperative complications following RPD-VR is increased when compared to OPD-VR. This is question cannot be currently answered.

As already reported for both renal and pancreatic robotic transplants [57, 58], the use e-PFTE for running vascular sutures is associated with several potential advantages. e-PFTE is a microporous vascular suture, mostly used in heart surgery, armed with a needle of the exact same size of the suture [59]. e-PTFE has no memory, good sliding properties [60], and elicits minimal tissue reaction [61]. When handled by robotic needle drivers, e-PTFE shows no loss in strength after repetitive manipulations, whereas polypropylene is weakened when touched three times at the same point [62]. In robotic sutures, maximal failure force of polypropylene is reduced by 35% as compared with 3% for ePTFE [63].

Following OPD with SMV/PV resection, benchmark rates of portal vein thrombosis and occlusion at hospital discharge are ≤ 14% and ≤ 4%, respectively [64]. A recent study demonstrates that the risk of thrombosis is higher in type 4 vein resection and reconstruction [65]. In RPD-VR reported rates of SMV/PV stenosis/thrombosis range between 7 and 9% [4, 43]. Therefore, the risk of SMV/PV thrombosis raises the important question on how to manage anticoagulation in these patients. Unfortunately, there is no agreed reply to this important question. The AHPBA guidelines for managing venous thromboembolism prophylaxis and anticoagulation for pancreatic surgery state that “*data for anticoagulation after reconstruction is inconclusive and support for perioperative chemoprophylaxis with pancreatic surgery is similarly limited*”. The final comment is:”*the recommendation of anticoagulation after vascular reconstruction in pancreatic resection is weak (grade of recommendation, weak 2B)*” [66]. In this study we have reported one partial SMV/PV thrombosis. In OPD, we have reported no SMV/PV occlusion with an incidence of non-occlusive thrombosis of 1.8% [12]. We speculate that this low rate of vascular thrombosis is the combined result of careful surgical technique plus standard anticoagulation prophylaxis. Clearly, more studies are needed to define the type of anticoagulant prophylaxis that should be used following pancreatoduodenectomy with SMV/PV resection and reconstruction.

In conclusion, the technique of RPD-VR requires some adaptations when compared to the open procedure. Here we have presented the tips and tricks implemented in 36 RPD-VR. Some of them were borrowed from OPD. Other technical details were imported from our robotic experience with renal and pancreatic transplantation. We suggest that surgeons willing to pursue RPD-VR should have a sound experience in OPD with vein resection and reconstruction. Hopefully, the tips and tricks provided in this study will facilitate safer implementation of RPD-VR.

## Supplementary Information

Below is the link to the electronic supplementary material.

Supplementary file1 (MP4 75123 kb)

Supplementary file2 (MP4 96918 kb)

Supplementary file3 (MP4 87007 kb)

Supplementary file4 (MP4 79545 kb)

## Abbreviations

e-PTFE: Expanded polytetrafluoroethylene
ISGPS: International study group of pancreatic surgery
LMWE: Low-molecular-weight heparin
MIPD: Minimally invasive pancreatoduodenectomy
OPD: Open pancreatoduodenectomy
RPD: Robotic pancreatoduodenectomy
RPD-VR: Robotic pancreatoduodenectomy with vein resection
SMA: Superior mesenteric artery
SMV: Superior mesenteric vein
SMV/PV: Superior mesenteric/portal vein
